# Supplementary material for: Engineering of Primary Human B cells with CRISPR/Cas9 Targeted Nuclease
Source: Sci Rep. 2018 Aug 14;8:12144. doi: 10.1038/s41598-018-30358-0 (PMC6092381; doi:10.1038/s41598-018-30358-0)
Supplement: Supplementary file 1 — Supplementary Figures [file 41598_2018_30358_MOESM1_ESM.pdf]

Engineering of Primary Human B cells with CRISPR/Cas9 Targeted Nuclease

Matthew J. Johnson[123], Kanut Laoharawee[123], Walker S. Lahr[123], Beau R. Webber[123], Branden S. Moriarity[123*]

[1]Department of Pediatrics, University of Minnesota, Minneapolis MN 55455, USA; [2]Center for Genomic Engineering, University of Minnesota, Minneapolis MN 55455, USA; [3]Masonic Cancer Center, University of Minnesota, Minneapolis MN 55455

*Corresponding Author: Branden S. Moriarity, Cancer and Cardiovascular Research Building, 2231 6th St. SE, University of Minnesota, Minneapolis, MN 55455, USA. Ph: +612 625 2226; Email: mori0164@umn.edu

Supplementary Figure 1

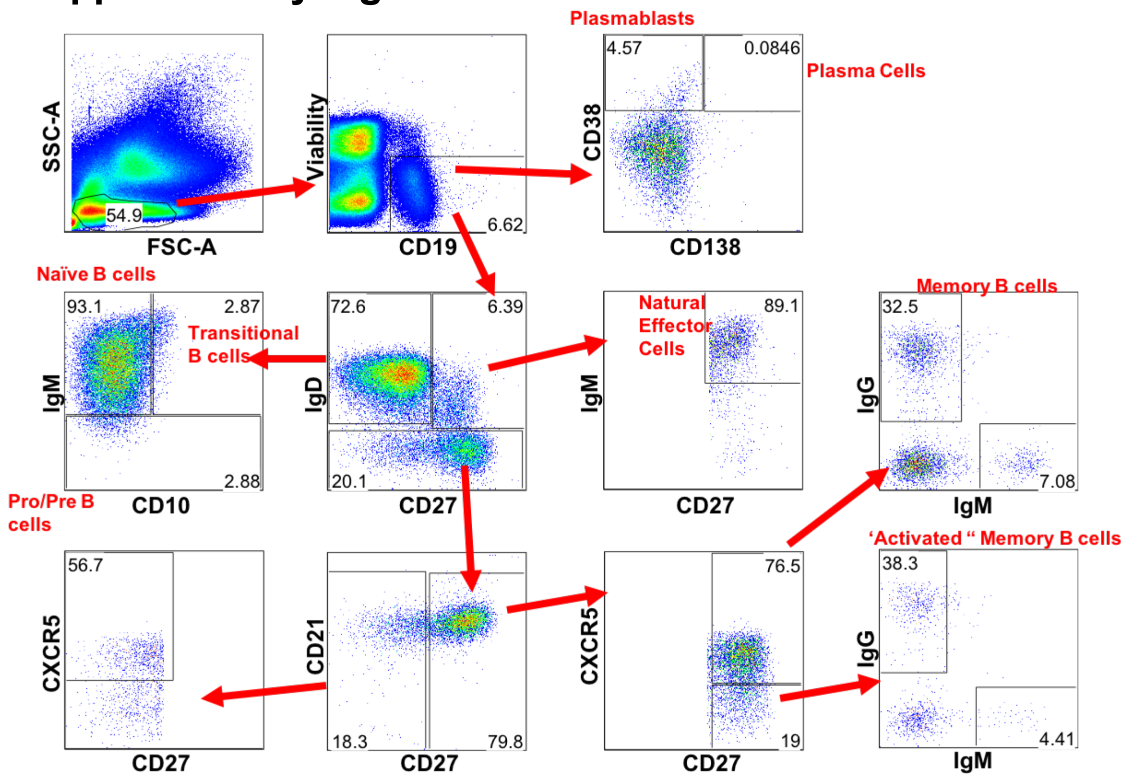

B cell subsets in the peripheral blood

Gating scheme used for the identification of B cell subsets by flow cytometry.

Supplementary Figure 2

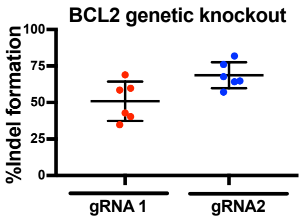

Knockout of BCL2

Percent indel formation with two different gRNAs targeting BCL2.

Supplementary Table 1

Specificity	Fluorophore	Clone	Vendor
CD10	BV711	HI10a	Biolegend
CD19	BUV395	SJ25C1	BD Horizon
CD20	PE/Dazzle 594	HIT2	Biolegend
CD21	BV650	B-ly4	BD OptiBuild
CD27	BV605	O323	Biolegend
CD38	BUV496	HIT2	BD Horizon
CD138	BV510	MI15	Biolegend
CXCR5	BV421	RF8B2	BD Horizon
IgD	PE	IA6-2	BD Pharmingen
IgG	APC	M1310G05	Biolegend
IgM	PE-Cy7	MHM-88	Biolegend

Flow cytometry antibodies

List of antibodies used for flow cytometry in this study

Unmodified, full-sized images of gels used to construct Figures 5C and 6C.

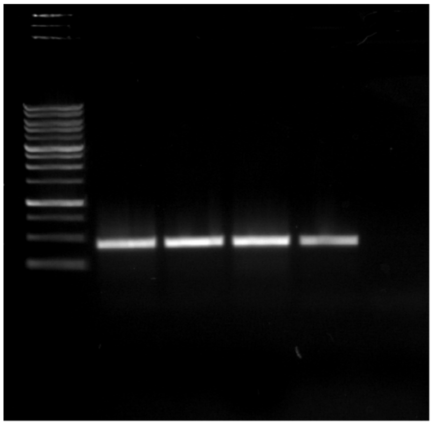

Figure 5C *left panel*

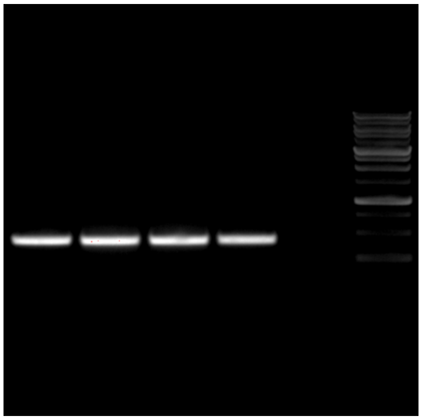

Figure 5C *central panel*

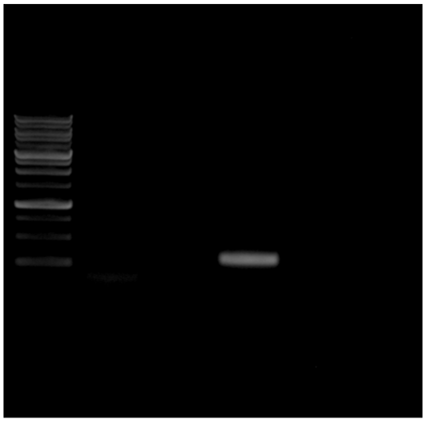

Figure 5C *right panel*

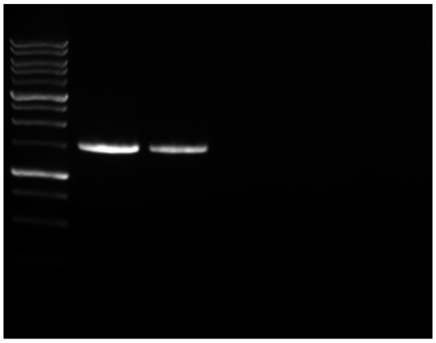

Figure 6C
